# Evaluation of salivary protein patterns among a rural population exposed and non-exposed to arsenic-contaminated drinking water in areas of Tucumán (Argentina): a pilot study

**DOI:** 10.1590/1678-7757-2020-0939

**Published:** 2021-09-03

**Authors:** Rosa Silvina GUBER, Mauricio GONZALEZ MAC DONALD, Mariano Nicolas ALEMAN, Maria Constanza LUCIARDI, Paula MENTZ, Alicia WIERNA, Carlos ANSONNAUD, Veronica GARCIA, Ana María ANSONNAUD, Analía SORIA

**Affiliations:** 1 Universidad Nacional de Tucumán Facultad de Bioquímica, Química y Farmacia Argentina Universidad Nacional de Tucumán, Facultad de Bioquímica, Química y Farmacia, Argentina.; 2 Universidad Nacional de Tucumán Facultad de Odontología Argentina Universidad Nacional de Tucumán, Facultad de Odontología, Argentina.

**Keywords:** Arsenic, Saliva, Oral, Malignant, SDS–PAGE

## Abstract

Arsenic contamination in the environment and groundwater is a major global public health problem. Several researchers suggest that the toxicity of arsenic could be related to oral cancer development, usually resulting from potentially malignant lesions. During pathological processes, salivary proteins suffer modifications, which could lead to the discovery of new biomarkers.

**Objective:**

To analyze the protein profile in human saliva samples from a rural population exposed to high levels of arsenic in drinking water and its association with potentially malignant lesions.

**Methodology:**

This observational, analytic and cross-sectional design included 121 patients from the state of Graneros (Tucumán, Argentina). Arsenic concentration in drinking water was determined and, according to the values obtained, individuals were divided into 2 groups: exposed group and non-exposed group. Saliva samples were obtained, and total protein concentration was measured by Bradford method. Finally, Laemmli SDS-polyacrylamide gel electrophoresis was conducted to obtain the protein profile.

**Results:**

Total protein concentration in saliva was lower in the exposed group than in the non-exposed group. Average areas of 20 and 42 KDa bands were significantly lower in exposed group than non-exposed group.

**Conclusion:**

Chronic intake of high arsenic concentrations in drinking water produces changes in the salivary protein profile, which is associated with the presence of potentially malignant lesions.

## Introduction

Arsenic is a metalloid found ubiquitously in the environment and its contamination of groundwater is considered a major global public health problem. It affects an estimated 140 million people in more than 50 countries, including the United States, Taiwan, Mexico, Mongolia, Argentina, India, Chile and others. Arsenic is present throughout the environment in its inorganic forms, and the level of exposure varies greatly by geographical location. The current World Health Organization (WHO) and Environmental Protection Agency (EPA) recommended limit for arsenic levels in drinking water is 10 μg/L.^1, 2^

Arsenic is considered as a class 1 human carcinogen by the International Agency for Research on Cancer (IARC) and the chronic exposure to arsenic is associated with chronic endemic regional hydroarsenicism (HACRE, in Spanish), which is characterized by skin lesions (leukoderma and/or keratosis), cardiovascular disease, neurotoxicity, type II diabetes, oral cancer (OC), and others.^3,4^

OC is a malignant neoplasia that arises on the lip or oral cavity. It is traditionally defined as a squamous cell carcinoma (OSCC), because 90% of cancers are histologically originated in the squamous cells.^5^ The main risk factors to the development of OC are tobacco, alcohol and betel nut intake, and human papillomavirus (HPV) infection. However, they do not explain the high OC incidence. Several researchers suggest that the toxicity of heavy metals present in the floor, such as lead, nickel, arsenic, and others, could be related to that. Despite the well-documented effect of arsenic exposure in the development of skin, lung and bladder cancer, evidence of a relationship with OC is scarce.^6-8^

OC presents precursor lesions that are not considered neoplasms, although they are related to a higher probability of developing into OSCC, known as potentially malignant lesions (PML). Those are: oral submucous fibrosis, erythroplakia, leukoplakia, chronic candidiasis, congenital dyskeratosis, lichen planus and actinic cheilitis.^9,10^

Saliva is a fluid produced and poured into the oral cavity by the salivary glands. After secretion into the oral cavity, it mixes with a serum transudate that emanates from the gingival sulcus. The singular contribution of gingival fluid allows markers to be present in the saliva which derive from the circulation.^11^ Proteins are another component of saliva that suffers modifications during pathological processes. These changes affect the protein profiles produced, which could lead to the discovery of new biomarkers and new approaches for diagnosis and detection of PML. For certain malignant diseases, there are markers that can be detected in saliva such as anti-p53 antibodies in patients with OSCC.^12^

This pilot study aimed to analyze the protein profile present in human saliva samples from a rural population exposed to high levels of arsenic in drinking water and its association with PML.

## Methodology

### Study design/patient selection

This observational, analytic and cross-sectional design included 121 patients of both sexes, from state of Graneros (Tucumán, Argentina), selected between May 2017 and December 2018.

Inclusion criteria were patients older than 35 years that lived in the residence for more than 10 years. Exclusion criteria were subjects with physical or mental disabilities, liver, pancreas and kidney diseases, anemias, muscular dystrophies, acute or chronic inflammatory processes (rheumatoid arthritis, lupus, diabetes), recent viral diseases, depression, narcotics or aspirins consumption and those undergoing chemotherapy or systemic radiotherapy.

All patients underwent a full clinical evaluation, including demographic, socioeconomic and personal data, time of residence in the region, smoking, alcohol and drug intake (medicines, natural herbs and others), family history of cancer, previous illnesses and type of drinking water supply. Also, all individuals underwent a gingival and periodontal clinical examination. Incisional biopsies were performed only on patients with any suspicious lesions to confirm clinical diagnosis. The biopsies were performed in the areas most prone to malignant transformation. Moreover, a saliva sample was obtained from each patient.

### Arsenic exposure assessment

Assistance units were installed in the work areas, where volunteers were received. Participants brought a sample of water from the home well to determine the arsenic concentration of the water they habitually consumed by the quantitative Gutzeit (silver diethyldithiocarbamate) colorimetric method.^13^ According to the values obtained, individuals were divided into 2 groups: exposed group (EG) with arsenic concentration higher than 0.05 mg/L in drinking water; and non-exposed group (NEG) with levels lower than 0.05 mg/L in drinking water (cutoffs established by the Argentine Food Code).^14^

### Biochemical parameters

To standardize the collection according to the circadian rhythm, unstimulated saliva samples were obtained after nighttime fasting, between 8 and 10 am, to prevent food ingestion, which would cause salivary secretion by increasing neurotransmitter activity due to taste and mastication stimuli. Before collection, patients washed their mouths with distilled water to remove any possible debris or contaminating material. They had to salivate for five minutes without swallowing and then expectorate into the sterile plastic container calibrated in milliliters until the desired amount (5 mL) was collected.^15^ Saliva samples were placed in the ice box in order to maintain the temperature between 2°C to 4°C. Protease Cocktail inhibitor (1 μL/mL of whole saliva) was added to the saliva immediately after collection to minimize protein degradation. Then, cold samples were centrifuged at 3000 rpm for 10 minutes to remove any unwanted particles; 500 µL of the supernatant was separated in Eppendorf and stored at -70°C until processing.

Determination of protein concentration in saliva samples by the Bradford method: some 300 μL of the supernatant was collected and an equal sample buffer volume was added. The mixture was placed in a water bath at 95°C for 10 minutes and protein concentration was determined by Bradford Reagent kit B6916 (Sigma-Aldrich, USA) following the manufacturer’s instructions. In total,10 μL of sample was added to Bradford’s solution (190 μL) and incubated 30 minutes at room temperature. The absorbance was measured in a spectrophotometer (VICTOR™ X – Multilabel Plate Reader, PerkinElmer, USA) at 595 nm wavelength and the data was processed with Gen5 Data Analysis Software (Biotek, USA). The detection range of the kit was 1-1.400 μg/mL protein.

Determination of protein profile: Unidimensional SDS-PAGE was performed following the technique described by Laemmli. The volume of each saliva sample containing 30 μg of total protein was estimated. The samples were then loaded onto 12.5% bis-tris-polyacrylamide gels with Tris base buffer (25 mM Tris base, 0.20 M glycine and 0.10% w/v SDS). A molecular weight marker pre-stained SDS-PAGE Standards, broad range (Bio Rad Life Science Laboratories, Hercules, CA, 1610320) was seeded with the samples from each gel to estimate the MW of the bands, and the samples from the NEG group were used as a reference. The electrophoretic run was performed under the following conditions: 150 volts constant for approximately 80 min. Finally, the gels were stained with Coomassie Brilliant Blue R- 250, faded with 30% methanol and 10% acetic acid in distilled water and digital images of the gels were acquired. The intensity of the protein profile bands was measured with Image Tool ver. 3.0 software (UTHSCSA, USA), which directly correlated them with their protein concentration.

### Statistical analysis

Statistical analysis was performed using the IBM SPSS Statistics ver. 25.0 (IBM Co., Armonk, NY, USA). Kolmogorov-Smirnov test was used to determine quantitative variables distribution. All data were expressed as frequency and percentage for categorical data and mean±standard deviation. Differences in study participants’ characteristics were compared across subgroups with chi-square test for categorical variables and Mann-Whitney’s U test for continuous variables, as appropriate. A p-value <0.05 was considered significant. For the statistical power estimating, the G*Power software ver. 3.1.9.6 (Franz Faul, University Kiel, Germany) was used.

### Ethical statement

This investigation protocol was approved by the Ethics Committee (RES Nº 7/2017) and all participants signed informed consent approved by institutional standards. The study was conducted in accordance with the declaration of Helsinki with good clinical practice as defined by the International Conference on Harmonization.

## Results

Table 1 shows demographic and social characteristics of the rural population studied. The mean age of the participants was 57.3±8.1 years. According to the education levels, around half were illiterate and the rest had only basic education. About 62% of participants reported low monthly income. Low skilled work concentrates the main economic activity and a large part of the population does not work. All subjects are considered vulnerable, since they have no access to the health system, so care and monitoring of oral-dental pathologies are infrequent.

**Table 1:** Demographic and social characteristics

Characteristcs		
**Participants (n)**		**121**
**Mean age, years (SD)**		**57.3±8.1**
	**%**	**95%CI**
**Gender**		
Male	71.9	63.9-79.9
Female	28.1	20.1-36.1
**Alphabetization**		
Incomplete basic education	44.6	35.7-53.5
Primary school	45.5	36.6-54.4
Secundary school	9.9	4.9-15.2
**Current marital status**		
Married	47.1	38.2-56.0
Unmarried	43.8	35.0-52.6
Unmarried cuple	9.1	4.0-14.2
**Monthly household income (US$)**		
<150	62.0	53.0-71.0
150-300	38.0	29.4-46.6
**Ocupation**		
Homemaker	28.1	20.1-36.1
Not working	37.2	28.6-45.8
Low skilled work	34.7	26.2-43.2

Homemaker: a person who manages the home. Not working: unemployer, retired. Low skill: manual labor, farmer, minicipal employer, etc.

**Figure 1:**
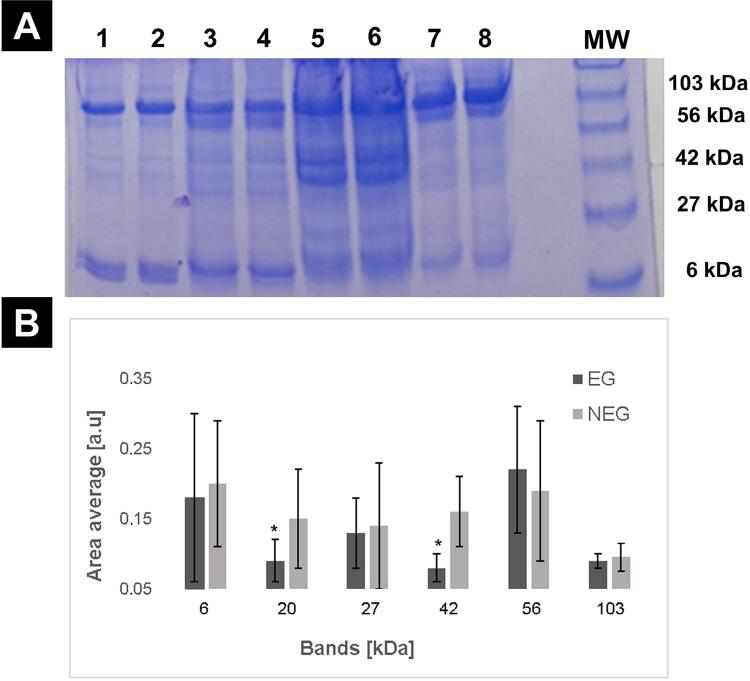
Effect of arsenic in drinking water on protein profile in saliva

Participants of this research were divided into two groups: EG (n=55) and NEG (n=66). Risk factors analysis for the development of PML (smoking, hazardous drinking and harmful drinking alcohol consumption) showed no significant differences between both groups (data not shown). Furthermore, patients in EG showed a lower total protein concentration in saliva than NEG (6.6±1.9 and 8.7±4.1, respectively). Table 2 shows the densitometric analysis (percentage and area average) of electrophoretic run in polyacrylamide gel ^16^. Average areas of 20 and 42 KDa bands were significantly lower in EG than NEG (p<0.01). Figure 1 shows an example of the different protein bands obtained with the SDS-PAGE electrophoresis technique.

**Table 2:** Protein profile in saliva samples

Protein fraction KDa	95%CI		Area average		p	b
	EG	NEG	EG	NEG		
6	100	84.8 (72.6-97.0)	0.18±0.12	0.20±0.09	0.161	0.26
20	16.7 (0-33.9)	15.1 (2.9-27.3)	0.09±0,03	0.15±0.07	0.01 *	0.99
27	27.5 (6.9-48.1)	21.2 (7.3-35.1)	0.13±0.05	0.14±0.9	0.082	0.11
42	32.9 (11.6-55.2)	30.3 (14.6-46.0)	0.08±0.02	0.16±0.05	0.012 *	1.00
56	100	100	0.22±0.09	0.19±0.10	0.235	0,39
103	27.5 (6,9-48.1)	27.3 (12.0-42.0)	0.09±0.01	0.095±0.02	0.073	0,39

EG: exposed group (n=55); NEG: non exposed group (n=66). * p<0.05 was considered significant (EG vs NEG)

A: Representative SDS-polyacrylamide gel electrophoresis results obtained from saliva samples of EG (1, 2, 3, 4, 7, and 8) and NEG (5, 6) groups. B: Densitometric analysis of the detected bands. The values were expressed as mean±SD. * p<0.05 was considered significant. EG: exposed group, NEG: non exposed group, MW: molecular weight.

**Figure 2:**
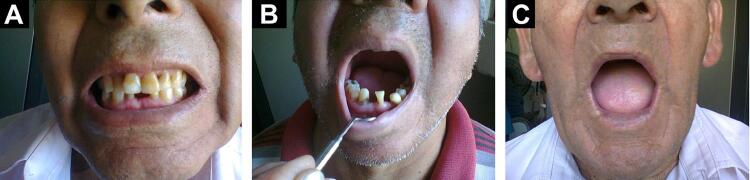
Photographs of oral pathologies detected in the exposed group

Oral pathologies in soft and hard tissue present in EG were chronic periodontitis (common cause of tooth loss), actinic cheilitis and stained teeth.^16^ Whereas, in NEG the pathologies found were biofilm–gingival, frictional keratosis and chronic periodontitis (Figure 2). Table 3 shows the main pathologies found in soft and hard tissues of the oral cavity.

A) stained teeth; B) chronic periodontitis; C) actinic cheilitis.

**Table 3:** Oral pathologies in soft and hard tissue

Pathologies	EG n (%)	NEG n (%)
Without pathologies	13 (23.63%)	18 (27.27%)
CP	11 (20.00%)	6 (9.10%)
AC	27 (49.09%)	0
AC + CP	4 (7.27%)	0
Dental biofilm–induced gingivitis	0	34 (51.51%)
FK	0	4 (6.06%)
FK + Dental biofilm–induced gingivitis	0	4 (6.06%)

EG: exposed group (n=55); NEG: non exposed group (n=66). CP: chronic periodontitis; AC: actinic cheilitis; FK: frictional keratosis.

Table 4 shows the correlation between modified protein bands, chronic periodontitis and actinic cheilitis in EG. These results indicate that actinic cheilitis correlates strongly with decreased protein bands of 20 and 42 KDa in the saliva samples analyzed. This decrease would not be affected by chronic periodontitis presence.

**Table 4:** Correlation between 20 kDa and 42 kDa bands between Actinic cheilitis and Chronic periodontitis

Pathologies	20 kDa bands				42 kDa bands			
	Undiminished	Diminished	p	b	Undiminished	Diminished	p	b
Without actinic cheilitis	18	8	0.01*	0.80	20	6	0.01*	0.99
With actinic cheilitis	10	19			7	22		
Without chronic periodontitis	24	18	0.10	0.48	23	19	0.13	0.37
With chronic periodontitis	4	9			4	9		

EG (n=55). * p<0.05 was considered significant.

## Discussion

Contamination of drinking water with arsenic in rural areas is a global public health problem, especially in Latin America. The effect of chronic exposure to this metalloid has been well studied and is related to different pathologies.^2^ However, evidence of the relationship between arsenic and oral pathology is scarce. In this pilot study, we evaluated the proteins present in human saliva samples in a Latin American rural population exposed to high levels of arsenic in drinking water and investigated the association of salivary proteins with the presence of PML.

Socio-demographic indicators showed that approximately 50% of the studied rural population is poor and the population does not work or has low-skilled jobs. These results emphasize the vulnerability of the subjects studied, which would justify their exposure to oral pathologies. Moreover, no significant differences were observed in the sociodemographic characteristics between the groups studied (EG and NEG). Similar characteristics were reported in other countries with poor rural populations.^17,18^ According to the 2010 national census of population, households and housing, state of Graneros has the highest number of unsatisfied basic needs (28%). Additionally, 35.3% of drinking water samples had arsenic levels exceeding those allowed by the Argentine Food Code, which is in accordance with the findings of other authors.^19,20^

Arsenic chronic consumption in drinking water can affect the salivary glands by modifying the concentration and expression of proteins that compose the saliva, which are essential for maintaining the health of oral tissues.

Some patient habits are recognized as risk factors to developing PML, of those, tobacco and alcohol consumption are the most important. Besides, their simultaneous consumption increases 10 to 20 times the risk of developing OC.^21^ In our study, both factors were found in equal proportion in EG and NEG groups.

The interesting results of this study were the total protein concentration decrease and the change in protein profile in saliva samples. There is no previous data available in literature about a decrease in saliva proteins due to chronic intake of arsenic in drinking water. However, other authors studied changes in saliva by the action of other agents. Actis, et al.^22^ (2006) reported a decrease of total protein content in saliva among young alcohol consumers. Awasthi^23^ (2017) informed significantly higher levels of protein in the saliva of patients with OSCC compared to individuals with PML and healthy controls. Likewise, total protein concentration in saliva from caries patients was significantly higher.^24,25^ These discrepancies could be explained by the different methodologies used, impeding a direct comparison.

Saliva proteins have been implicated as possible markers for diagnosis of different diseases such as oral, lung and breast cancers, cardiovascular diseases, and systemic disorders.^26,27^ Studies have not yet associated arsenic with PML.

Saliva is a fluid that contains a wide variety of proteins, and their identification depends on the method used, which explains the differences between numbers of proteins reported. Mucins, amylase, proline-rich proteins and secretory immunoglobulin A are the most abundant proteins in saliva. Electrophoresis separates proteins into multiple bands or fractions based on charge and size, which suggests that changes in the bands (particularly in cases of reduced banding) could be due to a modification in protein concentration of those with higher concentrations.^28,29^ In this pilot study, electrophoretic analysis of saliva samples showed lower concentration of 20 KDa and 42 KDa bands in EG.

Likewise, arsenic in all forms is carcinogenic to humans and effects of exposure to this metalloid vary according to the dose, route and duration of exposure, and individual’s genetics.^30^ Arsenic is a genotoxic and clastogenic agent that can cause damage of different classes and magnitudes in chromosomes. WHO defines PML as those tissues with altered morphology and a high predisposition to transform into cancer (>5%) compared to equivalent tissue of normal appearance, independently of their clinical or histological characteristics. In addition, it is a reversible state and does not necessarily imply cancer development. The main PML of the oral cavity are leukoplakia, erythroplakia, submucous fibrosis, lichen planus and actinic cheilitis.^31^ They are a pre-clinical phase of OC, which is highly prevalent both in men and in women. This is the reason why the National Cancer Control Strategy and Plan of Action have been developed with the objective of providing a universal service based on quality and timely care, trying to ensure that this issue is effectively addressed with evidence-based decision making.^32^ In the state of Cordoba (Argentina), a patient resident of rural arsenical area was documented to have multiple carcinomatous lesions located in the oral cavity.^33^ In the population studied, 50% of patients exposed to high levels of arsenic in drinking water were diagnosed with actinic cheilitis. They were reported to both work and live in rural areas. The synergism between exposure to As and agrochemicals (pesticides) could have multiplied the effect along the oral carcinogenesis process.^34^

This study suggests that the chronic intake of high arsenic concentrations in drinking water decreases protein saliva levels and changes in their profile. This is associated with a high percentage of potentially malignant lesions. Therefore, saliva analysis could be used as a diagnostic sample in these patients, due to their advantages compared to other samples, since its obtention is easy, safe, non-invasive, economic and better tolerated.

This preliminary study has some limitations; its cross-sectional design only allows association, but no causality. Since it is a pilot study, the results cannot be generalized. The sample size is relatively small, and some results cannot be applied indiscriminately. Therefore, further studies are needed to confirm the results, such as those aimed at establishing the relationship between protein concentration in saliva and arsenic levels in water; investigating markers of chronic exposure in hair and nails; and identifying altered proteins (20 and 42 kDa bands) by techniques used for the analysis of saliva as mass spectrometry or liquid chromatography.

## Conclusion

Chronic intake of high arsenic concentrations in drinking water produces changes in the salivary protein profile and this is associated with the presence of potentially malignant lesions.

## References

[1] - Sinha D, Prasad P. Health effects inflicted by chronic low-level arsenic contamination in groundwater: a global public health challenge. J Appl Toxicol. 2020;40(1):87-131. doi: 10.1002/jat.3823 10.1002/jat.3823 31273810

[2] - Litter MI, Ingallinella AM, Olmos V, Savio M, Difeo G, Botto L, et al. Arsenic in Argentina: occurrence, human health, legislation and determination. Sci Total Environ. 2019;676:756-66. doi: 10.1016/j.scitotenv.2019.04.262 10.1016/j.scitotenv.2019.04.262 31055207

[3] - Palma-Lara I, Martínez-Castillo M, Quintana-Pérez JC, Arellano-Mendoza MG, Tamay-Cach F, Valenzuela-Limón OL, et al. Arsenic exposure: a public health problem leading to several cancers. Regul Toxicol Pharmacol. 2020;110:104539. doi: 10.1016/j.yrtph.2019.104539 10.1016/j.yrtph.2019.104539 31765675

[4] - Huang HW, Lee CH, Yu HS. Arsenic-induced carcinogenesis and immune dysregulation. Int J Environ Res Public Health. 2019;16(15):2746. doi: 10.3390/ijerph16152746 10.3390/ijerph16152746 PMC6696092 31374811

[5] - Totan A, Imre Melescanu M, Miricescu D, Stanescu I, BencZe M, Radulescu R. Autophagy-a hidden but important actor on oral cancer scene. Int J Mol Sci. 2020;21(23):9325. doi: 10.3390/ijms21239325 10.3390/ijms21239325 PMC7729760 33297472

[6] - Candia J, Fernández A, Somarriva C, Horna-Campos O. Mortalidad por cáncer oral en Chile, 2002-2012 [Deaths due to oral cancer in Chile in the period 2002-2012]. Rev Med Chil. 2018;146(4):487-93. Spanish. doi: 10.4067/s0034-98872018000400487 10.4067/s0034-98872018000400487 29999124

[7] - Pal P, Halder A. Is there any role of arsenic toxicity in hpv related oral squamous cell carcinoma? Biol Trace Elem Res. 2019;188(2):274-83. doi: 10.1007/s12011-018-1419-6 10.1007/s12011-018-1419-6 29959645

[8] - Pal P, Raychowdhury R, Dolai TK, Roy S, Dastidar R, Halder A. Study of arsenic exposure in oral/oropharyngeal carcinoma in West Bengal. Int J Occup Med Environ Health. 2017;30(2):271-9. doi: 10.13075/ijomeh.1896.00806 10.13075/ijomeh.1896.00806 28366956

[9] - Speight PM, Khurram SA, Kujan O. Oral potentially malignant disorders: risk of progression to malignancy. Oral Surg Oral Med Oral Pathol Oral Radiol. 2018;125(6):612-27. doi: 10.1016/j.oooo.2017.12.011 10.1016/j.oooo.2017.12.011 29396319

[10] - Tilakaratne WM, Jayasinghe RD. Oral Potentially Malignant Disorders (OPMDs). In: Ranawaka RR, Kannangara AP, Karawita A, editors. Atlas of dermatoses in pigmented skin. Singapore: Springer; 2021. p. 879-902. doi: 10.1007/978-981-15-5483-4_44

[11] - Woźniak M, Paluszkiewicz C, Kwiatek WM. Saliva as a non-invasive material for early diagnosis. Acta Biochim Pol. 2019;66(4):383-8. doi: 10.18388/abp.2019_2762 10.18388/abp.2019_2762 31799813

[12] - Khurshid Z, Zafar MS, Khan RS, Najeeb S, Slowey PD, Rehman IU. Role of salivary biomarkers in oral cancer detection. Adv Clin Chem. 2018; 86:23-70. doi: 10.1016/bs.acc.2018.05.002 10.1016/bs.acc.2018.05.002 30144841

[13] - Arbab-Zavar MH, Chamsaz M, Heidari T. Speciation and analysis of arsenic (III) and arsenic(V) by electrochemical hydride generation spectrophotometric method. Anal Sci. 2010; 26(1):107-10. doi: 10.2116/analsci.26.107 10.2116/analsci.26.107 20065596

[14] 14 - Consultora de Aguas. Normas oficiales para la calidad del agua Argentina [Internet]. Buenos Aires: Consultora de Aguas; c2015 [cited 2019 May 4]. Available from: http://www.cdaguas.com.ar/pdf/aguas/24_Normas_oficiales.pdf

[15] - Song X, Yang X, Narayanan R, Shankar V, Ethiraj S, Wang X, et al. Oral squamous cell carcinoma diagnosed from saliva metabolic profiling. Proc Natl Acad Sci U S A. 2020; 117(28):16167-73. doi: 10.1073/pnas.2001395117 10.1073/pnas.2001395117 PMC7368296 32601197

[16] - Wetzel SL, Wollenberg J. Oral potentially malignant disorders. Dent Clin North Am. 2020;64(1):25-37. doi: 10.1016/j.cden.2019.08.004 10.1016/j.cden.2019.08.004 31735231

[17] - Sacoor C, Payne B, Augusto O, Vilanculo F, Nhacolo A, Vidler M, et al. Health and socio-demographic profile of women of reproductive age in rural communities of southern Mozambique. PLoS One. 2018;13(2):e0184249. doi: 10.1371/journal.pone.0184249 10.1371/journal.pone.0184249 PMC5796686 29394247

[18] - Rahman MA, Rahman A, Khan MZ, Renzaho AM. Human health risks and socio-economic perspectives of arsenic exposure in Bangladesh: a scoping review. Ecotoxicol Environ Saf. 2018;150:335-43. doi: 10.1016/j.ecoenv.2017.12.032 10.1016/j.ecoenv.2017.12.032 29304476

[19] - Gerstenfeld S, Jordán A, Calli R, Farías P, Malica J, Gómez Peña M, et al. Determinación de zonas de riesgo al agua arsenical y prevalencia de HACRE en Villa Belgrano, Tucumán, Argentina [Determination of zones exposed to Arsenical Water and CERHA prevalence in Villa Belgrano, Tucumán, Argentina]. Rev Argent Salud Publica. 2012;3(10)24-9. Spanish.

[20] - Guber RS, Tefaha L, Arias NN, Sandoval NG, Toledo R, Fernandez M, et al. Contenido de arsénico en el agua de consumo en Leales y Graneros (Provincia de Tucumán-Argentina). Acta Bioquim Cli n Latinoam. 2009;43(2):201-7.

[21] - Araya C. Diagnóstico precoz y prevención en cáncer de cavidad oral Early diagnosis and prevention in oral cavity cancer [Early diagnosis and prevention in oral cavity cancer]. Rev Med Clin Condes. 2018;29(4):411-8. Spanish. doi: 10.1016/j.rmclc.2018.06.008

[22] 22 - Actis AB, Simbrón A, Brunotto M, Gómez de Ferraris ME. Concentración de proteínas totales en saliva de jóvenes consumidores sociales de alcohol. Acta Odontol Venez [Internet]. 2006 [cited 2021 July 6];44(2):171-5. Spanish. Available from: http://ve.scielo.org/scielo.php?script=sci_arttext&pid=S0001-63652006000200004&lng=es.

[23] - Awasthi N. Role of salivary biomarkers in early detection of oral squamous cell carcinoma. Indian J Pathol Microbiol. 2017;60(4):464-8. doi: 10.4103/IJPM.IJPM_140_16 10.4103/IJPM.IJPM_140_16 29323056

[24] - Pyati SA, Naveen Kumar R, Kumar V, Praveen Kumar NH, Parveen Reddy KM. Salivary flow rate, pH, buffering capacity, total protein, oxidative stress and antioxidant capacity in children with and without dental caries. J Clin Pediatr Dent. 2018;42(6):445-9. doi: 10.17796/1053-4625-42.6.7 10.17796/1053-4625-42.6.7 30085875

[25] - Araujo HC, Nakamune AC, Garcia WG, Pessan JP, Antoniali C. Carious lesion severity induces higher antioxidant system activity and consequently reduces oxidative damage in children's saliva. Oxid Med Cell Longev. 2020;2020:3695683. doi: 10.1155/2020/3695683 10.1155/2020/3695683 PMC7008261 32089767

[26] - Castagnola M, Scarano E, Passali GC, Messana I, Cabras T, Iavarone F, et al. Salivary biomarkers and proteomics: future diagnostic and clinical utilities. Acta Otorhinolaryngol Ital. 2017;37(2):94-101. doi: 10.14639/0392-100X-1598 10.14639/0392-100X-1598 PMC5463528 28516971

[27] - Jain S, Paul S, Meena RN, Gangwar A, Panjwani U, Ahmad Y, et al. Saliva panel of protein candidates: A comprehensive study for assessing high altitude acclimatization. Nitric Oxide. 2020;95:1-11. doi: 10.1016/j.niox.2019.11.007 10.1016/j.niox.2019.11.007 31778801

[28] - Contreras-Aguilar MD, Vialaret J, Deville de Périère D, Escribano D, Lehmann S, Tecles F, et al. Variation of human salivary alpha-amylase proteoforms in three stimulation models. Clin Oral Investig. 2020;24(1):475-86. doi: 10.1007/s00784-019-03021-9 10.1007/s00784-019-03021-9 31388762

[29] - Welsh JA, Jenkins LM, Kepley J, Lyons GC, Moore DM, Traynor T, et al. High sensitivity protein gel electrophoresis label compatible with mass-spectrometry. biosensors (Basel). 2020;10(11):160. doi: 10.3390/bios10110160 10.3390/bios10110160 PMC7694097 33142797

[30] 30 - World Health Organization. Preventing disease through healthy environments: exposure to arsenic: a major public health concern [Internet]. Geneva: WHO; 2019 [cited 2021 July 6]. Available from: https://apps.who.int/iris/bitstream/handle/10665/329482/WHO-CED-PHE-EPE-19.4.1-eng.pdf?sequence=1&isAllowed=y

[31] - Yang EC, Tan MT, Schwarz RA, Richards-Kortum RR, Gillenwater AM, Vigneswaran N. Noninvasive diagnostic adjuncts for the evaluation of potentially premalignant oral epithelial lesions: current limitations and future directions. Oral Surg Oral Med Oral Pathol Oral Radiol. 2018;125(6):670-81. doi: 10.1016/j.oooo.2018.02.020 10.1016/j.oooo.2018.02.020 PMC6083875 29631985

[32] - Koo MM, Unger-Saldaña K, Mwaka AD, Corbex M, Ginsburg O, Walter F, et al. Conceptual framework to guide early diagnosis programs for symptomatic cancer as part of global cancer control. JCO Glob Oncol. 2021;7:35-45. doi: 10.1200/GO.20.00310 10.1200/GO.20.00310 PMC8081530 33405957

[33] - Carrica V. Carcinomatosis múltiple de localización bucal en un paciente de zona de Hidroarsenicismo Crónico Regional Endémico (HACRE) [Oral multiple carcinomatosis in a patient from an area with endemic regional chronica hydroarsenicism (ERCH)]. Rev Fac Cienc Med (Cordoba). 2006;63(2):20-4. Spanish. 17645042

[34] - Leonardi N, Gilligan GM, Panico RL. Oral squamous cell carcinoma associated with arsenic exposure: a case series from argentina. Int J Odontostomat. 2020;14(4):596-601. doi: 10.4067/S0718-381X2020000400596

